# Sequencing platform and library preparation choices impact viral metagenomes

**DOI:** 10.1186/1471-2164-14-320

**Published:** 2013-05-10

**Authors:** Sergei A Solonenko, J César Ignacio-Espinoza, Adriana Alberti, Corinne Cruaud, Steven Hallam, Kostas Konstantinidis, Gene Tyson, Patrick Wincker, Matthew B Sullivan

**Affiliations:** 1 Department of Ecology and Evolutionary Biology, University of Arizona, Tucson, AZ, USA; 2 Department of Molecular and Cellular Biology, University of Arizona, Tucson, AZ, USA; 3 CEA, DSV, IG, Genoscope, 2 rue Gaston Crémieux CP5706, Evry, Cedex, 91057, France; 4 Department of Microbiology and Immunology, University of British Columbia, Vancouver, BC, Canada; 5 Department of Civil and Environmental Engineering, Georgia Institute of Technology, Atlanta, GA, USA; 6 Austalian Center for Ecogenomics, University of Queensland, Brisbane, QLD, Australia

## Abstract

**Background:**

Microbes drive the biogeochemistry that fuels the planet. Microbial viruses modulate their hosts directly through mortality and horizontal gene transfer, and indirectly by re-programming host metabolisms during infection. However, our ability to study these virus-host interactions is limited by methods that are low-throughput and heavily reliant upon the subset of organisms that are in culture. One way forward are culture-independent metagenomic approaches, but these novel methods are rarely rigorously tested, especially for studies of environmental viruses, air microbiomes, extreme environment microbiology and other areas with constrained sample amounts. Here we perform replicated experiments to evaluate Roche 454, Illumina HiSeq, and Ion Torrent PGM sequencing and library preparation protocols on virus metagenomes generated from as little as 10pg of DNA.

**Results:**

Using %G + C content to compare metagenomes, we find that (i) metagenomes are highly replicable, (ii) some treatment effects are minimal, e.g., sequencing technology choice has 6-fold less impact than varying input DNA amount, and (iii) when restricted to a limited DNA concentration (<1μg), changing the amount of amplification produces little variation. These trends were also observed when examining the metagenomes for gene function and assembly performance, although the latter more closely aligned to sequencing effort and read length than preparation steps tested. Among Illumina library preparation options, transposon-based libraries diverged from all others and adaptor ligation was a critical step for optimizing sequencing yields.

**Conclusions:**

These data guide researchers in generating systematic, comparative datasets to understand complex ecosystems, and suggest that neither varied amplification nor sequencing platforms will deter such efforts.

## Background

Advances in sequencing technologies have revolutionized the life sciences. For example, ecology and evolution can now be examined across the tree of life [1], and at resolutions ranging from broad analyses (e.g., BGI’s 10,000 Microbial Genomes Project, http://ldl.genomics.cn/page/M-research.jsp) to focused investigation of population structure within particular species [2]. These analyses, however, center on genomes as the unit of interest and represent a “bottom-up approach” to exploring the diversity of life [3].

Concurrently, metagenomics provides a “top-down approach” for studying complex microbial assemblages in nature [3]. Recent reviews cover next generation sequencing applications [4-6], but rarely acknowledge the factors that generate quantitative data needed for metagenomics. For example, sequence quality evaluated across benchtop systems did not consider library preparation [7], and recommendations of amplification-free protocols that require >2 μg of DNA to minimize biases [8] are not meaningful for DNA-limited applications. There are also numerous sequencing platform options, though microbial metagenomes generated across commonly-used sequencing platforms only minimally differ in taxonomic distributions or contig assembly quality [9].

Some fields, such as viral ecology or microbial ecology of permafrost soils or the atmosphere, are routinely DNA-limited (<1 ng) and thus require optimization and quantitation assessment at each step in the metagenomic sample-to-sequence pipeline [10]. Towards this end, empirical data are now available to guide researchers in concentrating and purifying viruses [11,12] prior to DNA extraction. Once DNA is extracted, small yields require amplification to obtain enough material for sequencing. While whole genome amplification was an attractive option, it is now documented to result in non-quantitative metagenomes due to both stochastic [13] and systematic biases [14]. In contrast, linker-amplification-based libraries [15-17] provide a nearly quantitative alternative, even from sub-nanogram amounts of DNA [15]. Together these advances allowed the compilation of the first large-scale, systematically prepared comparative metagenomic dataset for quantitative viral ecology [18] with new tools and analytical platforms now emerging to handle such datasets [19,20]. Beyond viral ecology, these studies provide a roadmap for generating quantitative metagenomic datasets from any low (<100 ng) input DNA samples.

**Figure 1:**
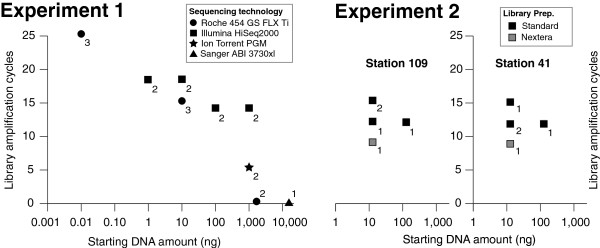
**Experimental design overview.** Library preparation treatments were done at varying levels of replication, as indicated by the numbers (1 to 3) next to each treatment. The number of amplification cycles (see y axis) includes those necessary to generate enough DNA for library preparation, but does not include the emPCR (454, Ion Torrent) or bridge (Illumina) amplification cycles used to build large enough populations of reads for nucleotide sequencing signal detection.

Here we expand upon these efforts to focus on the final steps in viral metagenomic sequencing (overview in Figure 1, and sequencing statistics summarized in Table 1). The first experiment evaluates co-varied input DNA and amplification cycle amounts, as well as sequencing platform choice on the resulting metagenomes. These data were derived from DNA extracted from a 1,080L Biosphere 2 Ocean viral concentrate and included small-insert metagenomes prepared from varied low-input DNA amounts (10 pg—100 ng) and amplification conditions for commonly used sequencing platforms (Illumina HiSeq2000, herein ‘Illumina’ and Roche 454 Titanium, herein ‘454’). Additionally, these low-input samples were complemented by standard input DNA(≥1,000ng) small-insert metagenomes to compare three sequencing platforms (Illumina, 454, Ion Torrent) and limited large-insert clone library Sanger end-sequencing (8,000ng fosmid library). The second experiment focuses on Illumina sequencing only. Here, viral DNA derived from two separate ocean samples (Tara Oceans [21] stations 41 and 109) was used to examine the effect of amplification conditions (e.g., cycle number) and input DNA amount independently, as well as compare standard Illumina libraries to transposon-based Nextera libraries [22].

**Table 1:** Summary statistics for all metagenomic libraries used in analysis

	**DNA source**	**Technology**	**Starting DNA (ng)**	**Library amplification (# cycles)**	**Replicates**	**Raw reads (millions)**	**Raw quality +/-SD (PHRED)**	**Raw length (bp)**	**Failed QC +/- SD (%)**
***Experiment 1***	Biosphere 2 Ocean	Illumina HiSeq 2000	1,000	14	2	65.5, 51.8	34.2 +/- 0.0	100 PE	29.9 +/- 0.5
			100	14	2	6.7, 0.3	33.8 +/- 0.2	100 PE	28.3 +/- 0.2 *
			10	18	2	2.5, 0	32	100 PE	31.9 *
			1	18	2	0, 0	0	0	0
		Roche 454 GS FLX	1,500	0	2	0.30, 0.38	32.5 +/- 0.7	408 +/- 11	15.4 +/- 0.4
			10	15 (LA)	3	0.91, 0.90, 0.85	32.8 +/- 0.8	377 +/- 15	31.5 +/- 4.0
			0.01	25 (LA)	3				
		Ion Torrent PGM 316 chip	1,000	5	2	2.3, 2.4	16.3 +/- 0.2	105 +/- 5	40.3 +/- 7.6
		ABI 3730xl	8,000	0	1	0.7	44.6	603	7.9
***Experiment 2***	Tara Oceans Station 41	Illumina HiSeq 2000	10	9 (N)	1	20.3	34.8	101 PE	36.3
			10	12	2	18.6, 31.3	34.2 +/- 0.2	101 PE	36.2 +/- 0.9
			10	15	1	15.4	34.3	101 PE	35.7
			100	12	1	17.7	34.6	101 PE	35.0
	Tara Oceans Station 109	Illumina HiSeq 2000	10	9 (N)	1	2.6	34.9	101 PE	35.4
			10	12	1	20.4	34.9	101 PE	34.3
			10	15	2	28.6, 16.2	34.4 +/- 0.5	101 PE	33.6 +/- 0.6
			100	12	1	16.7	34.8	101 PE	34.3

Starting DNA refers to the amount of pre-size selection DNA used in library construction; Library amplification abbreviations are LA = linker amplification and N = Nextera; Raw quality scores reported are PHRED scores; Raw length ‘PE’ denotes paired end reads. * denotes the successful 10ng library and one of the 100 ng libraries had an additional 40% of QC-passed reads that were lost due to removal of TruSeq adaptor sequence contaminants.

## Results

### Experiment 1: The impact of input DNA, amplification, and sequencing platform on metagenomes

#### Library success varies by sequencing protocol

As expected, the fosmid library and all 6 libraries made from ≥1,000 ng DNA were successful in generating sufficient DNA for sequencing regardless of sequencing platform (Table 1). Additionally, low DNA input libraries for 454 (linker-amplified [15] to obtain sufficient genetic material) were all successful, with highest read yields per ng of input DNA of any method (Additional file 1: Figure S1).

In contrast, Experiment 1 Illumina libraries constructed from low starting DNA amounts were less successful (Table 1). Specifically, 3 of 6 libraries, one 10ng and both 1ng libraries, failed library construction, even with the addition of carrier DNA and adaptor concentration adjustment to increase ligation efficiencies. Two of the remaining 3 low input DNA libraries, one 10ng and two 100ng, were sequenced, but yielded fewer and more variable numbers of reads and abundant adaptor sequence (see * in Table 1).

#### %G + C content variation within treatments is minimal

**Figure 2:**
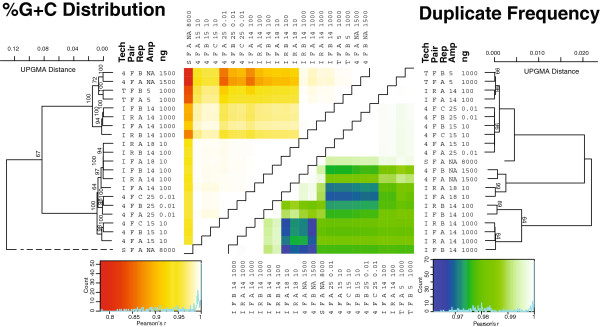
**%G + C and duplication plots for Experiment 1 metagenomes.** Heatmap coloring indicates the relative pairwise correlations (Pearson’s r) in the %G + C distributions (red-to-yellow) and duplicates (blue-to-green) where red and blue colors indicate the lowest levels of correlation, while white represents highly correlated data. The %G + C distribution correlations were UPGMA clustered with 100 bootstrap runs to indicate statistical support (only >60% support shown). Abbreviations are as follows: “Tech” is sequencing technology represented by 4 (454), T (Ion Torrent), I (Illumina), S (Sanger); “Pair” is the forward or reverse paired end sequence data; “Rep” is the arbitrarily labeled replicate ranging from two (**A** and **B**) to three (**A**, **B**, or **C**); “ng” is the nanograms of input DNA from which the viral metagenome was derived. The most reliable estimate of the true %G + C distribution is the unamplified 454 metagenomes. Relative to these, fosmid end sequences generated using Sanger sequencing were the most shifted toward high %G + C, while problematic <1000ng input DNA metagenomes were less shifted toward high %G + C, and reliable 1000ng Illumina metagenomes were only slightly shifted toward high %G + C.

The replicates’ read %G + C distributions were correlated using the Pearson product–moment correlation coefficient (Pearson’s r). There is little variation in %G + C across replicate libraries from any 454, Illumina, or Ion Torrent sequencing data – replicates have pairwise correlation values from 0.99 to 1 and cluster together >94% of the time (Figure 2). This indicates that, at least for the range of %G + C in this B2O sample, intra-replicate variation is minimal and therefore there is high power to detect statistically significant differences across treatments.

#### Input DNA amount, decision to amplify impact %G + C content

Hierarchical clustering of sample %G + C distribution correlations shows consistent differences. First, all ≥1,000 ng metagenomes cluster together 100% of the time (Figure 2). Of the treatments tested, input DNA most strongly impacts the resulting metagenomes, with ≥1,000 ng next-generation metagenomes clearly separated from the rest. Among these ≥1,000 ng samples, Illumina metagenomes have higher %G + C than 454 and Ion Torrent metagenomes (see Additional file 1: Figure S2 for example %G + C distribution plots), but differences between sequencing platforms are much less than differences between DNA inputs, with UPGMA branch length distances of 0.02 and 0.16, respectively (Figure 2). While of limited sampling, the largest shift towards higher %G + C sequences (Pearson’s r <0.8) was in the fosmid library relative to the unamplified libraries (Figure 2, Additional file 1: Figure S3).

Among the <1,000ng metagenomes, there are minimal differences between platforms and the only supported relationship, with bootstraps greater than the intra-replicate 94% value, was the clustering of Illumina 100ng samples with Illumina 10 ng samples (Figure 2). This implies that, among amplified metagenomes, the degree of amplification and sequencing platform choice only minimally impact the resulting metagenomes. The fact that these diversely prepared metagenomes were nearly indistinguishable by %G + C distribution metrics (Pearson’s r values >0.99, Figure 2) is promising for comparability of amplified metagenomes across sequencing platforms.

#### Duplicate reads uncorrelated with any single variable

Duplicates in metagenomes are derived from either naturally occurring duplicates in genomes and communities, or artificial duplicates generated during 454’s emPCR step or at some unknown point in Illumina preparations that is inconsistent across replicate libraries [23,24].

Here, hierarchical clustering of duplicate frequencies (Figure 2) and raw duplicate distributions, normalized to metagenome size (Additional file 1: Figure S3), suggest a pattern of three duplication groups. The first, composed of unamplified 454 and 10ng Illumina metagenomes, contains intermediate levels of duplication (14.6 to 42.7%) and few high-frequency (>10 fold) duplicate reads (0.06 to 5.1%). The second cluster, composed of most Illumina metagenomes, has an intermediate level of duplication (27.1 to 37.3%), but also an excess of highly duplicated reads (10.4 to 15.6%). The third includes the amplified 454 metagenomes, both Ion Torrent metagenomes, and the poorly amplified 100ng Illumina library, all of which have few duplicate reads (0.9 to 16.6%) and very few high-frequency duplicate reads (0.0005 to 0.9%) (Additional file 1: Figure S4). However, these deep internal nodes lacked support, with bootstraps less than the intra-replicate 90% value, and duplication frequencies do not obviously correlate to any single metagenome category (e.g., technology, amplification, DNA amount, or paired end).

Some duplicate sequences may be real. For example, one 100bp sequence is overrepresented in multiple libraries including 1,000ng Illumina (0.14% of the total reads), Ion Torrent (0.006%), and unamplified 454 (0.36%) libraries. Artificial duplicate frequency correlations (see Online Methods) match overall duplicate frequencies for all treatments except a single 10ng, poorly-amplified, adapter-containing Illumina library (Additional file 1: Figure S5-7), where 40% of the reads were predominantly artificial, high frequency duplicates (Additional file 1: Figure S8 and S9).

#### Gene function and read assembly parallel %G + C findings

**Figure 3:**
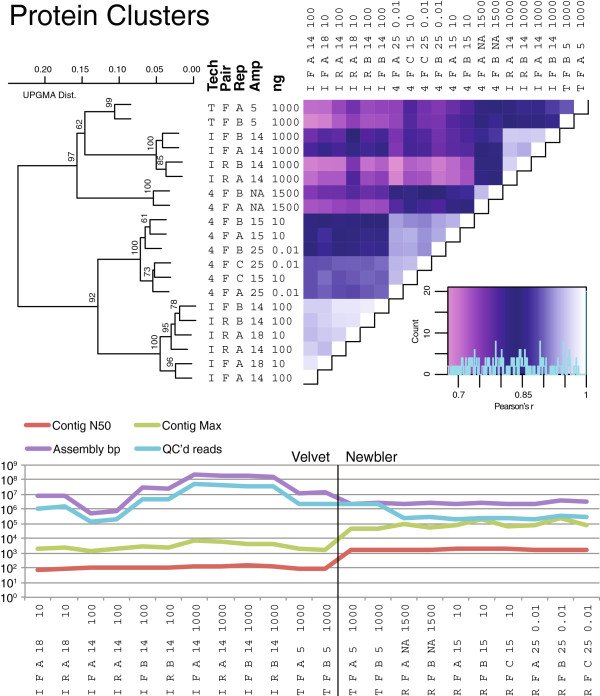
**Protein cluster functional analysis and assembly statistics for Experiment 1 metagenomes.** Metagenomic reads were mapped to POV protein clusters (see text) and hit frequencies were used to produce pairwise correlation heat maps. Details as described in Figure 2, including bootstrap analysis of statistical support for correlations across metagenomes. Assembly performance of each sample across the dataset was evaluated using metrics of n50 and maximum contig size, as well as the number of reads and base pairs that were assembled. Note that inferior assembly performance was restricted to samples with reduced read yields. Lastly, the Newbler assembler yielded larger contigs and smaller total assemblies when compared to Velvet assembly of the same Ion Torrent dataset.

To evaluate variations in gene function, metagenomic reads were compared to an expansive database of marine virus protein sequences (>456K protein clusters derived from over 6M reads from 32 diverse pelagic ocean virus communities [18]). As is common for viral metagenomes (reviewed in ref. [18]) only 3—7% of the reads mapped to protein clusters without self-clustering. However, the resulting gene frequency patterns were well-supported and mirror patterns observed in the above %G + C analyses (Figure 3A). Replicate metagenomes were most similar (pairwise r-values >0.95), while the biggest difference was between metagenomes prepared from ≥1,000 ng of starting DNA and those prepared from 100ng or less (r-values <0.8). Within these two large clusters, sequencing technology choice contributed additional, but minor, divergences (r-values 0.8—0.9). Notably, these protein cluster pairwise r-values are lower than those for either %G + C or duplicate frequency. This likely reflects increased analytical resolution, as 1,500 protein clusters correlated per metagenome in the function analysis, while only 50 or 10 bins were resolved in the %G + C and duplicate analyses, respectively.

Finally, assembly experiments (see Methods, Figure 3B) revealed that total assembly size positively correlated to the number of reads used in assembly. In turn, the maximum and N50 contig sizes were relatively insensitive to increased read numbers in the larger datasets. This was true for both k-mer and overlap-based assembly algorithms (see Methods).

### Experiment 2: The independent effects of input DNA and library amplification on Illumina-sequenced metagenomes

#### Low input DNA library success improved with optimization

**Figure 4:**
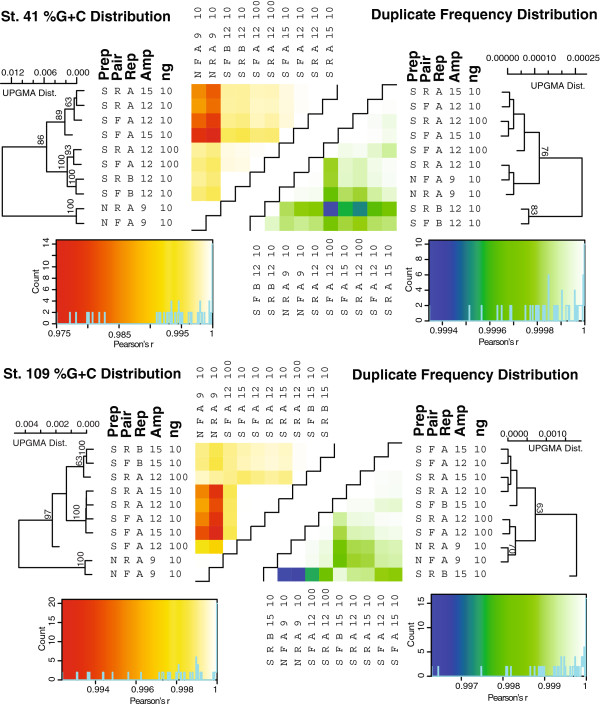
**%G + C and duplication plots for Experiment 2 metagenomes.** Details as described in Figure 2, including bootstrap analysis of statistical support for correlations across metagenomes. UPGMA clustering bootstrap support >60% shown only.

In contrast to Experiment 1, all 10 Experiment 2 Illumina libraries (eight 10ng and two 100ng libraries) were successful. Replicate libraries did not cluster together consistently, but this reflected the extremely minimal variance across the replicates rather than poor replication (Figure 4, note reduced axis scales relative to Figure 2).

#### Transposon-based library preparation slightly impacts %G + C

**Figure 5:**
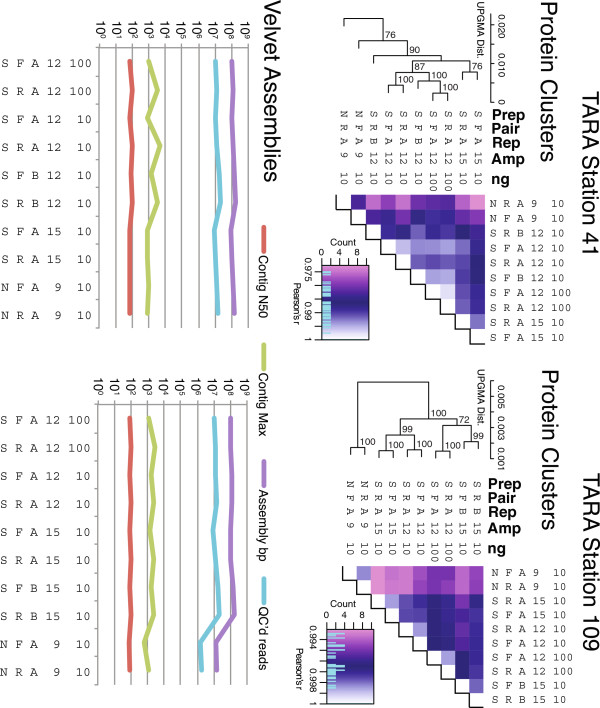
**Protein cluster functional analysis and assembly statistics for Illumina-sequenced Experiment 2 metagenomes.** Note that one metagenome from Station 109 DNA yielded significantly fewer reads and thus had a lower total assembly size. Details as described in Figure 3, including bootstrap analysis of statistical support for correlations across metagenomes.

In both Tara Oceans station 41 and 109 datasets, the amount of input DNA (10 or 100 ng) and amplification (12 or 15 cycles) resulted in less variation than was observed in replicate library preparations (Figure 4). The only exception was transposon-based libraries, which diverged from the relatively invariant standard Illumina libraries. For all samples, duplicate frequencies varied as much between as within treatments (Figure 4) and much less duplication was observed in Experiment 2 than 1. The dendrogram topology observed in pairwise %G + C analyses was recovered in analyses of function (Figure 5A), but not assembly (Figure 5B), where the transposon-based treatment for the Station 109 sample produced many fewer reads than other metagenomes.

## Discussion

Replication is fundamental to rigorous experimental design [25], but it is only now becoming financially possible for metagenomic studies [26,27]. Here we examined replicate metagenomes across varied DNA input amounts, library preparation procedures, and sequencing platforms.

### Low input DNA library success depends on adaptor ligation

While all ≥1,000 ng DNA libraries were successful, environmental samples, particularly for viruses, routinely yield <1ng of DNA [15]. Libraries constructed from ≤100 ng DNA were successful using the linker-amplification protocol for 454 [15], but Illumina libraries failed or were low-quality for Experiment 1, but not Experiment 2. Two separate protocols were used – both optimized for recovery from column purification steps [28], but employed different template:adaptor ratios in ligation [29]. Specifically, Experiment 1 used 170:1, while Experiment 2 used 22:1 for 10ng starting DNA. Thus low DNA libraries require adjusted adaptor:template ratios during ligation (see Genoscope protocol for guidelines).

### Presence of library amplification drives bias

Two amplification reactions are common in metagenomic sample preparations. The first, library amplification, increases input DNA to balance library preparation losses from purification, size selection, and quality titrations [8]. This adaptor-mediated amplification step is used for limiting DNA for 454 (15—25 cycles [15]), but is routinely employed in Ion Torrent (5 cycles) and Illumina (12—16 cycles) to enrich for correctly ligated adaptors. This step can alter overall library %G + C [15,17,30]. The second amplification step is specific to the sequencing technology (e.g., emPCR in 454 or Ion Torrent, bridge amplification in Illumina) and used for improving signal detection. This step should not alter overall library %G + C, but can artificially over-represent sequences [23,24].

In this study, two libraries received no library amplification: unamplified 454 and fosmid libraries. Fosmids had elevated %G + C, which is ascribed to a cloning bias [26]. Among the remaining libraries, we expected a low %G + C shift due to the adaptor-mediated amplification step, commonly attributed to inhibitory effects of high %G + C DNA secondary structures, either during library amplification [30] or downstream emPCR [31]. However, these trends were not observed: in Experiment 1, the 454 unamplified and amplified Illumina 1,000 ng libraries correlate well with one another (r-values > 0.99), but poorly (r-values < 0.9) with the amplified (18 cycles) 10ng Illumina libraries. This difference appears to be driven by reduced low %G + C reads relative to the ≥1,000 ng libra ries, which may implicate low input DNA libraries as more sensitive to loss of low %G + C reads either during gel extraction heat steps [32] or preferential fragmentation through heating [33]. A possible improvement over gel extraction is Sage Science’s Pippin Prep (tested with 65ng of DNA, see Figure 2B in ref. [15]), which avoids heating. Heat during fragmentation is avoidable with Covaris acoustic shearing. Both techniques also minimize contamination, which is crucial for DNA-limited libraries.

While amplified ≤100 ng metagenomes displayed different %G + C distributions from ≥1,000 ng metagenomes, the amount of amplification only minimally impacts the resulting metagenomes. This was true in Experiment 1, where starting DNA amount and amplification cycling co-varied, as well as Experiment 2, where these parameters were independent. Fragment competition resulting from cycling conditions is thought to select for higher %G + C and shorter fragments, thus linker-mediated amplification protocols employ tight sizing conditions and %G + C optimized PCR conditions [30]. Such careful library construction can produce minimally biased (<1.5-fold %G + C variation) viral metagenomes from sub-nanogram amounts of DNA [10,15]. The %G + C patterns observed in the current larger-scale study were also paralleled in functional analyses (protein cluster mapping) and assembly performance. This suggests that systematically prepared linker-amplified metagenomes derived from variable input DNA amounts are quantitatively comparable.

Some caution is warranted for high-throughput transposon-based library preparation options like Nextera. Specifically, Experiment 2 revealed that standard libraries prepared from limiting DNA and under varied conditions were relatively invariant, whereas the transposon-based protocol led to divergent %G + C and protein cluster profiles for metagenomes from both stations. While these deviations were statistically significant (90% bootstrap clustering in Figures 4 and 5), they were minor in magnitude relative to other treatment effects observed here. Such a %G + C bias in Nextera library preps is not entirely surprising as previous work demonstrated reduced coverage in both high and low %G + C regions of virus genomes [34], presumably due to non-random transposition. Evaluation of new transposition methods should be considered if their eventual products require strictly unbiased representation of input DNA.

Finally, while not investigated here, polymerases used in amplification can alter metagenomes. Phi29 polymerase, for example, leads to stochastic and systematic biases that can impact resulting coverage [13], while some high-fidelity polymerases (e.g., TAKARA) enrich for rare sequences and others (e.g., PfuTurbo) do not [11,15]. In Experiment 1, the ≥1,000 ng libraries only minimally differed from each other despite the fact that they employ different polymerases across sequencing platforms. These polymerase-specific effects would depend on protocol particulars (e.g., PCR cycler settings and additives) [17,30] and the underlying %G + C distribution (particularly for <20% or >80% G + C fragments) of the DNA to be amplified. Future work to determine the impact of polymerase choice empirically on metagenomes derived from a wider range of %G + C than those employed here would be informative.

### Duplicates vary by input DNA, amplification, technology

Duplicated reads are problematic in quantitative applications as they can be real or artificial [23,24,35,36]. Here, Experiment 1’s true distribution of duplicates is presumably represented by the first cluster (includes unamplified 454 libraries), except the artificial duplicates discussed below. By comparison, metagenomes from the second cluster contained highly duplicated artificial reads that reduced library complexity during amplification. The last cluster, which included amplified 454, as well as one Illumina and two Ion Torrent metagenomes, had low levels of duplication. For the 454 libraries, this could be due to the diversifying effects of the linker amplification process [15], but it is harder to explain this trend in the Ion Torrent metagenomes or find a process that ties low library amplification in the 100ng Illumina metagenome to lower duplication levels. Artificial duplicates in Illumina libraries were only an issue in the problematic 10ng library, where 40% of the reads were high-frequency, predominantly artificial duplicates. Further study is required to determine mechanisms that generate artificial duplicates in Illumina data.

### Sequencing technologies produce comparable output

While the metagenomes here were derived from three very different ocean viral communities, the range of %G + C was not extreme. Given that, sequencing technology is not a major factor impacting ocean viral metagenomes, which is consistent with previous microbial metagenomic studies [9]. However, read length can influence many downstream applications, from assembly efforts to functional identification of genes [37,38]. Of widely used next-generation technologies, 454 currently has the longest read length of 800bp, with paired-end Illumina capable of 250 + bp [7]. However, emerging nanopore technologies are likely to be truly transformative [39]. Details are not yet public, but these technologies promise longer reads, direct observation of fragment sequences, and minimal library preparation enabling low input DNA applications.

## Conclusions

As we strive for systematic and quantitative analyses of complex environments, a thorough understanding of empirically-documented biases in methods is critical. Here we demonstrate that while sequencing platform choice and degree of amplification have little impact on resulting metagenomes, presence of amplification and starting DNA amounts do influence library success and composition. Our findings are critical both for the interpretation of systematic comparisons of DNA-limited community metagenomes, as well as for novel methods of studying virus-host interactions [40-42] that generate small amounts of DNA. Notably, however, high replicability observed here might have been aided by diluting the initial concentrated DNA sample, and potential inhibitors, to obtain ‘low input DNA’ samples. Consideration should be made of the impact of inhibitors on low input DNA samples, particularly when amplification steps are needed for sample preparation.

Given current findings, unamplified libraries are best when DNA is not limiting (>2 ug) [43] while sequencing platform choice minimally impacts quantitative representation in the resulting metagenomes. When DNA is limiting, as in viral community samples or microbial communities of permafrost soils or air samples, specific recommendations for quantitative metagenomics are as follows. Low input DNA (1—100 ng) libraries can utilize either a linker-amplified protocol [15] optimized for the appropriate sequencing technology of choice [10] or, for Illumina sequencing, standard library preparations where adaptor:template ratios are carefully controlled. For samples with ultra-low DNA yields (<1 ng), it is best not to risk failure in standard library preparations and to use instead a sequencing technology optimized linker-amplified protocol. Future research directions include developing a mechanistic understanding of the non-intuitive, but replicable differences in linker-amplified metagenomes, as well as improving understanding of polymerase impacts and developing empirical datasets for a broader range of %G + C samples.

## Methods

### Source DNAs and sample preparation details

#### Experimental protocol availability

All detailed protocols are listed by name, and are documented and available at http://eebweb.arizona.edu/Faculty/mbsulli/protocols.htm*.*

Briefly, FeCl-precipitated viral concentrates were obtained from 0.2μm filtered seawater collected from the man-made Biosphere 2 Ocean in December 2010, as well as Stations 41 (Indian Ocean, 14°34.572 N 70°1 E, deep chlorophyll maximum) and 109 (south Pacific Ocean, 1°58.286 N 84°26.772 W, deep chlorophyll maximum) of the Tara Oceans expedition on March 30^th^, 2010, and May 12^th^, 2011, respectively. The viral concentrate from the former was purified using both CsCl and DNase, while only DNAse was used for the latter.

#### DNA Source for B2O metagenomes (Biosphere 2 Ocean)

The B2 Ocean environment is host to a stable microbial community, as measurements of microbial phyletic frequencies are consistent across samples taken a year apart (Additional file 2). FeCl precipitation [12] was used to concentrate viruses from 1,080L of 0.2 μm filtered seawater, which were then DNase I treated [11] to remove free DNA, cesium chloride purified to remove microbial contaminants (dsDNA viral band was pulled 1.4—1.52 g/ml [11]), and further concentrated to 4 mL using an Amicon 30KDa filter. The final yield was 1.26 × 10^12^ SYBR-stained virus particles. DNA was extracted using the Wizard Prep DNA Purification system (Promega, cat# A7211 and A7181).

#### DNA Source for TARA metagenomes

20—60L seawater was collected and filtered for two TARA Oceans [21] stations using the protocol described above. These samples yielded 690 ng (station 41) and 950 ng (station 109) of DNA, using the Wizard Prep DNA Purification system. Starting DNA amounts of 10 and 100 ng were used in Illumina sequencing library construction as described in the Genoscope protocol (Genoscope Illumina protocol).

#### 454 Library Prep (Sullivan lab)

The linker amplification protocol was used to generate amplicon libraries for 454 sequencing, as well as amplification-free libraries, as previously described [15]. Briefly, genomic DNA was Covaris-sheared, unidirectionally ligated to an adaptor, and amplified using adaptor-specific primers using 15 to 25 amplification cycles, depending on the starting DNA amount (a description of the amount of cycling and relationship to input DNA were documented previously [15]). Following the addition of barcodes, sequencing libraries were ligated to 454-specific adaptors.

#### Fosmid Library Prep (Hallam lab)

8μg of B2O viral DNA was used in large-insert fosmid library construction using the Epicentre CopyControl Fosmid Library Production Kit (CCFOS110) as previously described [44]. A total of 17 384-well plates of clones were picked, and 384 fosmids were sequenced bi-directionally with Sanger sequencing.

#### Ion Torrent Library Prep (University of Arizona Genomics Core)

2μg of B2O viral DNA was used for sequencing library preparation following the Ion Fragment Library Kit User Guide (Rev July 2011), loaded onto beads, emPCR-ed, then sequenced using the 316 chip on the Ion Torrent PGM.

#### Illumina Library Prep for B2O metagenomes (Emory Genomics Core)

DNA samples were Covaris-sheared and size-selected to 300—600bp using SPRI Size Selection chemistry, enrichment amplified using Phusion DNA polymerase according to starting amount of DNA (14—18 cycles), and paired end sequenced. Two libraries starting with 1ng of DNA failed to amplify to sufficient amounts, even with the use of a carrier DNA protocol (Emory carrier DNA protocol). One 10 ng library experienced the same problem, and was not sequenced. The libraries were multiplexed on two sequencing lanes, with one replicate of each starting amount library present together on each lane.

#### Illumina Library Prep for TARA metagenomes (Genoscope)

DNA samples were Covaris-sheared and size selected to 160—180bp, amplified according to starting amount of DNA (9—15 cycles) and paired-end sequenced. Several modifications of the standard Illumina protocol [32] were introduced in order to minimize losses of ultra-low DNA amounts. The low-fragment-size shearing settings, coupled with Ampure beads to remove very short fragments, ensured the recovery of appropriately sized fragments without the need for gel sizing. The Pfx Platinum polymerase was used to increase amplification efficiency and thus decrease the number of total library amplification cycles. During ligation, proper adaptor ratios were chosen to correspond to 2—3 fold more adaptor ends than fragment ends in the working ligation reaction (Genoscope Illumina protocol). Transposon-based Nextera libraries were prepared per manufacturer’s instructions using the Illumina compatible Nextera DNA Sample Prep Kit (Epicentre Biotechnologies, cat#GA09115).

### Bioinformatics methods

#### Script availability

All custom scripts are listed by name and available at http://code.google.com/p/tmpl/*.*

#### Sequencing data

All metagenomic sequences are publically available through the CAMERA portal at http://camera.calit2.net/ [CAMERA**:** CAM_P_00001027]. 454 and Ion Torrent data, provided by UAGC, were delivered in .sff format and converted for downstream processing to FASTA and QUAL formats using sffinfo (roche454 v2.6) and then to FASTQ format using BioPerl 1.6.1. B2 Ocean Illumina data, by Emory Genomics Core, and TARA Oceans Illumina data, by Genoscope, were provided in FASTQ format. Each library was examined for raw quality using FastQC (v0.9, downloaded Aug 2012 from http://www.bioinformatics.babraham.ac.uk/projects/fastqc/) and Fastx_Toolkit (v0.0.13 downloaded Feb 2010 from http://hannonlab.cshl.edu/fastx_toolkit/). The FastQC report was the source of duplication data used in the figures. Adapter sequences were detected in two metagenomes (I1A18N10 and I1A14N100) through the overrepresented sequences functionality of FastQC. The fastx_toolkit utility ‘fastx_clipper’ was used with the –C option to remove all reads matching the above adapter motif from the forward paired end reads, removing approximately 40% of the reads that passed QC in each of these libraries.

#### Quality control

Next, procedures for quality control were established to remove suspect sequence data, either by filtering whole reads or trimming reads in accordance with known sequencing technology artifacts. For 454 and Ion Torrent data, whole-read filtering was used, as is common for metagenomics [11,15,45,46] (Additional file 1: Figure S10). In contrast, because Illumina errors are localized to particular parts of a read [47,48], these data were trimmed using a threshold predicted quality score to remove suspect regions of the read at both the 3’ and 5’ ends using DynamicTrim.pl, part of the SolexaQA package [49] (Additional file 1: Figure S11). After QC steps, 69—85% of the 454 reads remained, compared to 60% for Ion Torrent and 63—74% for Illumina (Table 1). The fastx_toolkit software package was also used to remove Illumina reads under 50bp, while the 454 and Ion Torrent reads were cleaned using a custom pipeline [18]. This processing ensured that the data analyzed would be analogous to that used for metagenomic inference. FastQC and Fastx_Toolkit were used to check the QC process of each metagenome.

#### %G + C analytics

The mean read %G + C was chosen as the focus of our analysis, rather than the %G + C of sequence subsets of a read or the larger genome regions from which the read fragment originated, since mean fragment %G + C the best predictor of GC bias [50]. QC-ed reads were processed using the BioPerl 1.6.1 script bp_gc_calc.pl to obtain average %G + C values for each read. Given the large read length differences across these libraries (90bp to 350bp), only the first 50bp of each read are used in all %G + C distribution analyses to match the shortest QC-ed Illumina data, while normalizing for read length. Reads were truncated to 50bp using fastx_toolkit and processed with bp_gc_calc.pl. Phage metagenomic reads were cut into non-overlapping 50bp fragments using a bash script and also processed with bp_gc_calc.pl.

#### Statistical analysis and figures

R 2.14.1 (http://www.R-project.org/) was used to run a custom script, 0.02gc.R, which calculated frequencies of reads in 2% G + C bins for each metagenome. Pearsons’s r pairwise correlation values were calculated using the cov() function, and heatmap figures were generated using the heatmap.2() function found in the gplots library (http://CRAN.R-project.org/package=gplots). Lastly, bootstrapped UPGMA clustering values for each node were obtained using the pvclust() function in the pvclust library (http://CRAN.R-project.org/package=pvclust), with pairwise distances calculated from Pearson’s correlation values and hierarchical clustering done using the “average” method.

#### Duplicate analyses

Duplication levels were assessed in raw reads by counting the occurrence of duplicates only in the starting 50bp of each read using the FastQC duplication level utility output, normalized to total metagenome size to reflect relative frequencies. Artificial duplicates were defined as those with identical starts and >95% identity throughout the read, and were detected using CD-HIT-454 [51] and CD-HIT-DUP [52] with default parameters.

#### Protein cluster analyses

Functional differences within and between metagenomes were assessed in Experiment 1 by mapping metagenomic reads to the Pacific Ocean Virome database [18]. The hit frequencies of the 1,500 protein clusters that were most abundant across all metagenomes were then used to obtain pairwise correlation values. A range of 3—7% of the metagenomic reads mapped to these POV PCs, while the ‘top 1,500 PCs’ subsample represented >99% of the data that mapped. Because the Experiment 1 dataset represented a large diversity of read lengths, greatly impacting inference capacity [38], the dataset was normalized to assess sequencing platform biases rather than read length impacts as follows: (i) the longer Ion Torrent and 454 reads were trimmed to 100bp, and (ii) only reads ≥100 bp were used from Illumina data.

#### Assembly analyses

The short reads derived from Illumina and Ion Torrent data were assembled using Velvet v 1.2.03 [53] using default parameters across a range of kmer sizes (23, 27, 31bp), but only 31-mer data are reported as kmer size did not impact assemblies. The longer 454 reads were assembled using GS De Novo Assembler v2.6 (http://my454.com/products/analysis-software/index.asp) with default parameters.

## Competing interests

The authors declare that they have no competing interests.

## Authors’ contributions

SAS and MBS conceived the project and designed the experiments with contributions from AA, SH, KK, GT and PW. AA and CC performed experiments. SAS and JCIE collected and analyzed the results. SAS, MBS, SH, KK, GT wrote the manuscript. All authors read and approved the final manuscript.

## Supplementary Material

Additional file 1: Figures S1-S11 A log-log plot of all B2 Ocean metagenome read yields per starting DNA amount (**Figure S1**). % G + C histogram of several ‘problematic’ and ‘reliable’ libraries, and GC distribution of full dsDNA bacteriophage genomes for reference (**Figure S2**). %G + C distribution differences between whole-read mean % G + C in unamplified 454 metagenome, in green, and Sanger-sequenced fosmid library, in blue, shows a shift toward high %G + C in the fosmid library (**Figure S3**). Duplicate frequencies in Experiment 1 metagenomes (**Figure S4**). Heatmap of Pearson’s r pairwise correlation values for artificial duplicate frequencies, as detected using CD-HIT-454 for 454 and Ion Torrent data and CD-HIT-DUP for Illumina data (**Figure S5**). CD-HIT-454 artificial duplicate frequencies in Experiment 1 metagenomes generated using 454 and Ion Torrent sequencing (**Figure S6**). Duplicate frequency minus artificial duplicate frequency for Experiment 1 CD-HIT-454 –processed metagenomes (**Figure S7**). CD-HIT-DUP artificial duplicate frequencies in Experiment 1 Illumina metagenomes (**Figure S8**). Duplicate frequency minus artificial duplicate frequency for Experiment 1 CD-HIT-DUP –processed metagenomes (**Figure S9**). Ion Torrent QC length distribution (**Figure S10**). Methods for Trimming Illumina Reads (**Figure S11**). Click here for file

Additional file 2 **Pyrotag data for microbial composition of Biosphere 2 Ocean in Nov 2008 and Sep 2009.** The Biosphere 2 Ocean was the source of the DNA sample used in Experiment 1 metagenomes. The distribution of microbial phyla in the B2 Ocean community appears stable across two samples taken a year apart. Click here for file
