# Supplementary material for: Sequencing platform and library preparation choices impact viral metagenomes
Source: BMC Genomics. 2013 May 10;14:320. doi: 10.1186/1471-2164-14-320 (PMC3655917; doi:10.1186/1471-2164-14-320)
Supplement: Additional file 1: Figures S1-S11 — A log-log plot of all B2 Ocean metagenome read yields per starting DNA amount (Figure S1). % G + C histogram of several ‘problematic’ and ‘reliable’ libraries, and GC distribution of full dsDNA bacteriophage genomes for reference (Figure S2). %G + C distribution differences between whole-read mean % G + C in unamplified 454 metagenome, in green, and Sanger-sequenced fosmid library, in blue, shows a shift toward high %G + C in the fosmid library (Figure S3). Duplicate frequencies in Experiment 1 metagenomes (Figure S4). Heatmap of Pearson’s r pairwise correlation values for artificial duplicate frequencies, as detected using CD-HIT-454 for 454 and Ion Torrent data and CD-HIT-DUP for Illumina data (Figure S5). CD-HIT-454 artificial duplicate frequencies in Experiment 1 metagenomes generated using 454 and Ion Torrent sequencing (Figure S6). Duplicate frequency minus artificial duplicate frequency for Experiment 1 CD-HIT-454 –processed metagenomes (Figure S7). CD-HIT-DUP artificial duplicate frequencies in Experiment 1 Illumina metagenomes (Figure S8). Duplicate frequency minus artificial duplicate frequency for Experiment 1 CD-HIT-DUP –processed metagenomes (Figure S9). Ion Torrent QC length distribution (Figure S10). Methods for Trimming Illumina Reads (Figure S11). [file 1471-2164-14-320-S1.docx]

**Figure S1:** A log-log plot of all B2 Ocean metagenome read yields per starting DNA amount. The X-axis denotes starting DNA amount; the Y axis is the number of reads in each metagenome, normalized to starting DNA amount. The three metagenomes in the top left, giving most reads per ng of input DNA, were amplified using the Linker Amplification protocol.

**Figure S2: %**G+C histogram of several ‘problematic’ and ‘reliable’ libraries, and GC distribution of full dsDNA bacteriophage genomes for reference.

1. **Reliable metagenome and problematic metagenome.** Reliable 1000ng Illumina metagenome in blue, problematic 100ng Illumina metagenome in green. Pearson’s correlation r value of 0.95.
2. **Two reliable metagenomes**: Illumina 1000ng in blue, 454 1500ng in green. Pearson’s correlation r value of 0.99.
3. **Reference GC distribution within order Caudovirales.** Myoviridae family is in blue, Siphoviridae in green, and Podoviridae in red. Genomic sequences accessed from NCBI April 2012.

**Figure S3:** %G+C distribution differences between whole-read mean %G+C in unamplified 454 metagenome, in green, and Sanger-sequenced fosmid library, in blue, shows a shift toward high %G+C in the fosmid library.

**Figure S4:** Duplicate frequencies in Experiment 1 metagenomes. Calculated as exact duplicates over the first 50 bp of each read only.

**Figure S5:** Heatmap of Pearson’s r pairwise correlation values for artificial duplicate frequencies, as detected using CD-HIT-454 for 454 and Ion Torrent data and CD-HIT-DUP for Illumina data. Note the separate keys provided for CD-HIT-454 data and CD-HIT-DUP data.

**Figure S6**: CD-HIT-454 artificial duplicate frequencies in Experiment 1 metagenomes generated using 454 and Ion Torrent sequencing.

**Figure S7**: Duplicate frequency minus artificial duplicate frequency for Experiment 1 CD-HIT-454 –processed metagenomes.

**Figure S8**: CD-HIT-DUP artificial duplicate frequencies in Experiment 1 Illumina metagenomes.

**Figure S9**: Duplicate frequency minus artificial duplicate frequency for Experiment 1 CD-HIT-DUP –processed metagenomes.

**Figure S10**: Ion Torrent QC length distribution**.** Raw reads span a wide range of read lengths. After quality score and read length filtering, reads that pass QC have a much tighter length distribution. Reads that failed the quality filter only, <2 SD below mean read quality score, are distributed across the whole range of read lengths, as can be seen by comparing plot of Passed reads to the plot of reads that either Passed or Failed Quality filtering. Reads that failed the ambiguous nucleotide ‘N’ filter were very few in number, as seen by comparing the Raw read frequency plot to the plot of reads that either Passed or Failed Quality or Length filtering, but not N filtering.

**Figure S11**: Methods for Trimming Illumina Reads. DynamicTrim.pl trimming, used on Illumina data in this paper, finds the longest contiguous segment above a PHRED threshold score of 20, and trims off everything else. For example, read 2 is trimmed down to the region from bp 15 to bp 35. This plot also shows an alternative metric, BWA (lighter lines), which trims reads only at the 3’ end at the location of maximum of the BWA score (very light lines). The BWA score increases along a read as long as each bp PHRED score is over the threshold score of 20. The BWA metric keeps an excess of low-quality data compared to the DynamicTrim.pl procedure.

**Figure S1**

**Figure S2**

**
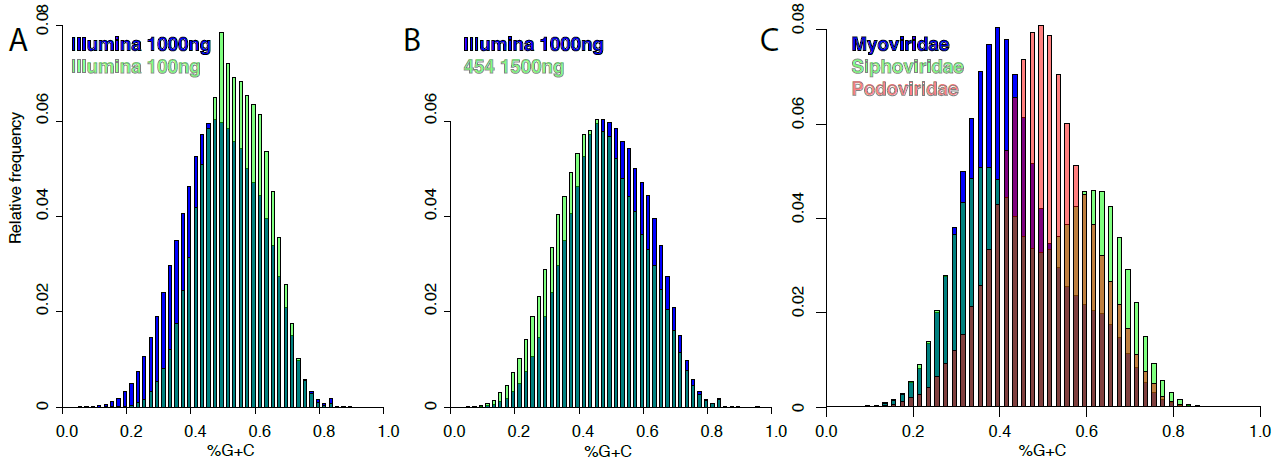
**

**Figure S3**

**
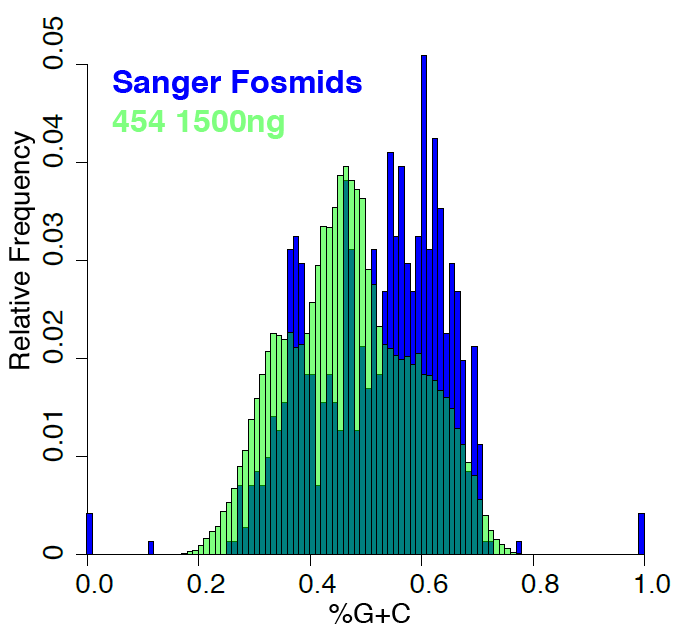
**

**Figure S4**

**Figure S5.**

**
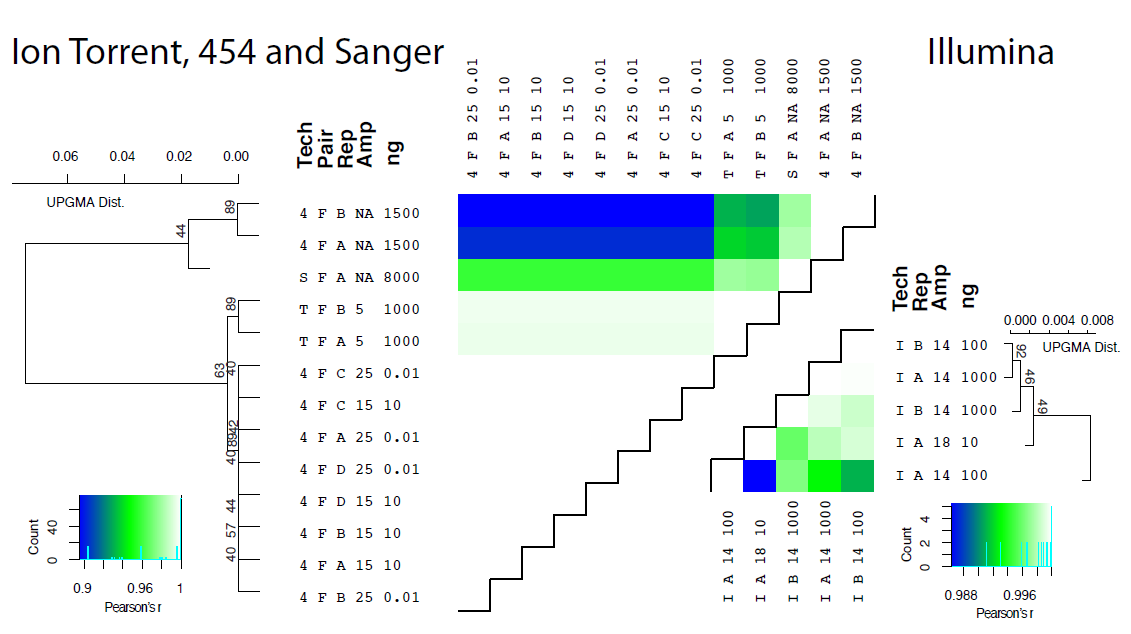
**

**Figure S6.**

**Figure S7.**

**Figure S8.**

**Figure S9.**

**Figure S10.**

**Figure S11.**
